# Early effects of the normal birth action plan on mode of birth, maternal preference, and cesarean indications in a private hospital setting in Türkiye

**DOI:** 10.3389/fmed.2026.1775492

**Published:** 2026-04-14

**Authors:** Seniye Burcu Torumtay Alic

**Affiliations:** Department of Gynecology and Obstetrics, Faculty of Medicine, Hitit University, Çorum, >Türkiye

**Keywords:** cesarean section, fear of childbirth, health systems, maternal autonomy, maternal request, mode of birth, normal birth action plan, obstetric policy

## Abstract

**Background:**

Cesarean section (CS) rates have risen globally to unprecedented levels, becoming a major public health concern. Türkiye has one of the highest CS rates among OECD countries, reaching approximately 57%, far above World Health Organization recommendations. In response, the Ministry of Health introduced the Normal Birth Action Plan (NBAP) in October 2024 to promote vaginal birth (VB) and reduce medically unnecessary CS. However, the real-world impact of NBAP in private hospitals—where maternal preference, fear of childbirth, institutional dynamics, and medico-legal concerns strongly influence mode of birth—remains unclear.

**Objective:**

To evaluate the early effects of NBAP on mode of birth distribution in a private hospital and to examine CS indications, maternal preference, and the role of NBAP-related counseling.

**Methods:**

This retrospective single-centre study included 2,226 births between October 2023 and October 2025, comparing 1-year pre-NBAP and post-NBAP periods. Maternal age, mode of birth, CS type, CS indication, and NBAP counseling status were analyzed. Categorical variables were compared using chi-square tests, with *p* < 0.05 considered statistically significant.

**Results:**

Mean maternal age was 27.37 ± 5.04 years (range: 18–43). CS rates decreased from 89% in the pre-NBAP period (1215/1365) to 81% in the post-NBAP period (698/861), representing a reduction in CS rate from 89% to 81%, alongside a decrease in absolute CS numbers, which should be interpreted in the context of reduced total births. VB increased from 11% to 19% (an 8%-point increase). CS attributed to maternal request or fear of VB declined from 26.1% to 17%. Mode of birth and CS indications differed significantly between periods (*p* < 0.001). Similar trends were observed across age groups (18–30 vs. ≥31 years). Monthly analyses showed a gradual increase in VB following NBAP implementation. Total births decreased by approximately 37% during the post-NBAP period, suggesting additional contextual or demographic influences.

**Conclusion:**

In this private hospital cohort, the post-NBAP period was temporally associated with a reduction in cesarean births and an increase in vaginal births, along with a decrease in CS attributed to maternal request or fear of VB. However, given the retrospective design and concurrent changes in birth volume, causality cannot be inferred. Multicentre studies with longer follow-up are required to clarify these associations.

## Introduction

The rapid increase in cesarean section (CS) rates worldwide over the past two decades has become a major public health concern with significant implications for maternal and neonatal health. The World Health Organization (WHO) reports that CS rates exceeding 10–15% do not reduce maternal or neonatal mortality; on the contrary, medically unnecessary cesarean deliveries increase the risk of complications (1). Despite this, CS rates have surpassed 50% in many countries, particularly in private healthcare institutions, and in some regions the majority of births are conducted as planned cesarean deliveries (2).

Similarly, in Türkiye, CS rates are well above the OECD averages (3, 4). Recent analyses based on the Robson classification system have further highlighted the contribution of specific obstetric groups to rising CS rates in Türkiye (5). According to data from the Ministry of Health, the national CS rate is approximately 57%, corresponding to nearly four times the upper limit recommended by the WHO. This high rate is not solely attributable to obstetric indications but is also associated with sociocultural, psychological, and institutional factors (6, 7).

Recent studies have demonstrated that fear of childbirth, concerns about labor pain, a perceived loss of control, and previous negative birth experiences play a significant role in women's requests for cesarean delivery (8, 9). Fear of childbirth is reported to be particularly pronounced among nulliparous women and can often be reduced through antenatal counseling or the development of individualized birth plans (10, 11). Nevertheless, cesarean preference is not merely an individual choice; it is shaped by multilayered factors, including communication gaps within the healthcare system, guidance from midwives and physicians, legal concerns, and prevailing societal perceptions of childbirth (2, 8).

In Türkiye, the Normal Birth Action Plan (NBAP) was implemented by the Ministry of Health in October 2024 with the aim of reducing CS rates and promoting vaginal birth (VB) (6). The plan includes multifaceted components such as the expansion of antenatal education programs, the establishment of mother-friendly hospital standards, the strengthening of midwifery services, the improvement of informed consent processes, and the monitoring of CS rates (12). While prioritizing safe childbirth, this action plan also aims to provide expectant mothers with physical comfort, privacy, and psychological support during the birth experience (6, 12).

However, childbirth preferences in private hospitals are shaped by different dynamics compared with public hospitals. In private healthcare settings, the active participation of pregnant women in decision-making regarding the mode of delivery is also considered an indicator of service satisfaction (7). Therefore, evaluating the impact of the NBAP requires consideration not only of statistical outcomes but also of patient preferences, levels of information, and clinical decision-making processes.

Private and public hospitals differ substantially in patient demographics, expectations, and care dynamics. Private settings often involve higher maternal autonomy, increased medico-legal concerns, and greater influence of patient preference on clinical decision-making, all of which may contribute to higher CS rates.

The aim of this study is to evaluate the temporal changes associated with the implementation of the NBAP on the distribution of delivery modes in a private hospital setting in Türkiye. By considering women's preferences regarding mode of birth, the study also explores the institutional, economic, and clinical dimensions related to this policy. The findings aim to contribute to understanding how national childbirth policies are reflected in clinical practice and to inform future strategies.

## Patients and methods

This study was designed as a single-centre, retrospective, before–after observational study evaluating births occurring before and after the implementation of the NBAP in a private hospital setting. The study protocol was approved by the Ethics Committee of Hitit University Faculty of Medicine (Decision No: 2025-214).

A total of 2,226 women who delivered between October 2, 2023, and October 2, 2025 were included. Based on October 2, 2024, the nationwide implementation date of the NBAP, the study period was divided into two phases: the pre-NBAP period (October 2, 2023–October 1, 2024) and the post-NBAP period (October 2, 2024–October 2, 2025). All births occurring at the study centre during these two consecutive 1-year periods were evaluated.

Inclusion criteria were: women who had live births at the study hospital; gestational age between 37 and 42 weeks; availability of complete medical records including mode of birth and indication; receipt or non-receipt of NBAP-related counseling; singleton or multiple pregnancies; and documented fetal presentation. Exclusion criteria included pregnancies complicated by fetal anomalies, intrauterine fetal demise, preterm births (< 37 weeks), incomplete or inaccurate medical records, and births performed at other institutions.

Data were extracted from the hospital electronic medical record system and included maternal age, fetal presentation, mode of birth, CS indication, elective and emergency CS, and NBAP counseling status. Distributions of modes of birth before and after NBAP implementation were compared. Temporal changes in VB and CS rates, CS subtypes, the influence of fetal presentation on mode of birth, changes in CS indications, and associations between NBAP counseling and mode of birth were analyzed.

Elective CS was defined as a planned cesarean delivery performed before the onset of labor, regardless of whether the indication was medical or maternal request.

NBAP implementation included antenatal counseling, increased awareness among healthcare staff, institutional monitoring of CS rates, and policy-driven recommendations. These components were gradually integrated into clinical practice following the national launch of the NBAP in October 2024.

The impact of fetal presentation on delivery outcomes was evaluated within the category of CS indications, particularly under abnormal presentation indications.

NBAP counseling status was determined based on documentation in the electronic medical records. However, variability in recording practices may have affected the accuracy of classification and should be considered when interpreting the results.

Maternal age was categorized using a cut-off value of 30 years. Although advanced maternal age is traditionally defined as ≥35 years, previous studies have shown that obstetric intervention rates begin to increase from the early thirties. Therefore, a 30-year threshold was selected to provide a clinically meaningful and statistically sensitive stratification of maternal age (13, 14).

This study is reported in accordance with the Strengthening the Reporting of Observational Studies in Epidemiology (STROBE) guidelines.

## Statistical analysis

Statistical analyses were performed using SPSS version 25 (IBM Corp., Armonk, NY, USA). Continuous variables were presented as mean ± standard deviation (SD) and minimum–maximum values. Categorical variables were summarized as frequencies and percentages.

Associations between NBAP period (pre vs. post) and mode of birth, CS subtype, and CS indications were assessed using the chi-square (χ^2^) test. Continuity correction was applied for 2 × 2 contingency tables, and the Pearson chi-square test was used for analyses involving multiple categories. As expected cell counts were ≥5, test assumptions were met. A *p*-value < 0.05 was considered statistically significant. The strength of associations was quantified using Phi (Φ) and Cramer's *V* coefficients as measures of effect size.

## Results

A total of 2,262 deliveries were identified during the study period. Thirty-six patients were excluded due to missing or incomplete data or delivery at another hospital. Consequently, 2,226 women were included in the final analysis.

The mean maternal age was 27.37 ± 5.04 years (range: 18–43). There were 1,365 births in the pre-NBAP period and 861 births in the post-NBAP period.

In the pre-NBAP period, 1,215 births (89%) were delivered by CS and 150 (11%) by VB. In the post-NBAP period, 698 births (81%) were CS and 163 (19%) were VB.

Regarding CS subtype, 460 cases (37.9%) were primary CS and 755 (62.1%) were secondary CS in the pre-NBAP period. In the post-NBAP period, 306 cases (43.8%) were primary CS and 392 cases (56.2%) were secondary CS.

The most frequent CS indications in both periods were previous uterine surgery/previous CS, maternal request or fear of VB, and cephalopelvic disproportion.

A statistically significant association was found between mode of birth and NBAP period (Pearson χ^2^ = 27.562; *p* < 0.001; Cramer's *V* = 0.111). A significant association was also observed for CS subtype (Pearson χ^2^ = 6.602; *p* = 0.010; Cramer's *V* = 0.059). In addition, the distribution of CS indications differed significantly between the two periods (Pearson χ^2^ = 56.449; *p* < 0.001; Cramer's *V* = 0.172; Table 1).

**Table 1:** Maternal characteristics and distribution of delivery modes before and after the NBAP.

Variables	Pre-NBAP (*n* = 1,365)	Post-NBAP (*n* = 861)	*p*-value
Age, mean ±*SD* (min–max)	27.09 ± 5.37 (18–43)	27.81 ± 4.52 (18–42)	0.084
Mode of delivery, *n* (%)			
Cesarean section (CS)	1,215 (89)	698 (81)	< 0.001
Vaginal birth (VB)	150 (11)	163 (19)	
CS subtype, *n* (%)			
Primary	460 (37.9)	306 (43.8)	0.010
Secondary	755 (62.1)	392 (56.2)	
CS indication, *n* (%)			
Previous uterine surgery/CS	460 (37.8)	306 (43.8)	< 0.001
Prolonged labor	89 (7.3)	35 (5)	
Cephalopelvic disproportion	212 (17.4)	112 (16)	
Fetal distress	15 (1.2)	29 (4.2)	
Abnormal presentation (breech/face/ transverse)	59 (4.9)	50 (7.2)	
Gestational hypertension/ preeclampsia	19 (1.6)	19 (2.7)	
Twin pregnancy	12 (1)	6 (0.9)	
Macrosomia	32 (2.6)	17 (2.4)	
Placental abnormalities (previa/abruption)	0	5 (0.7)	
Other (maternal CS request/fear of VB)	317 (26.1)	119 (17)	

Age-stratified analyses (18–30 vs. ≥30 years) showed similar patterns in both periods. In the post-NBAP period, CS rates decreased and VB rates increased in both age groups. However, no statistically significant association was found between maternal age group and mode of birth (pre-NBAP: χ^2^ = 2.795; *p* = 0.095; post-NBAP: χ^2^ = 1.212; *p* = 0.271). Similarly, no significant association was observed between maternal age group and CS subtype or CS indications in either period (*p* > 0.05).

Although counseling related to NBAP was documented in some patient records, data were insufficient for robust statistical analysis due to variability in documentation practices.

**Figure 1:**
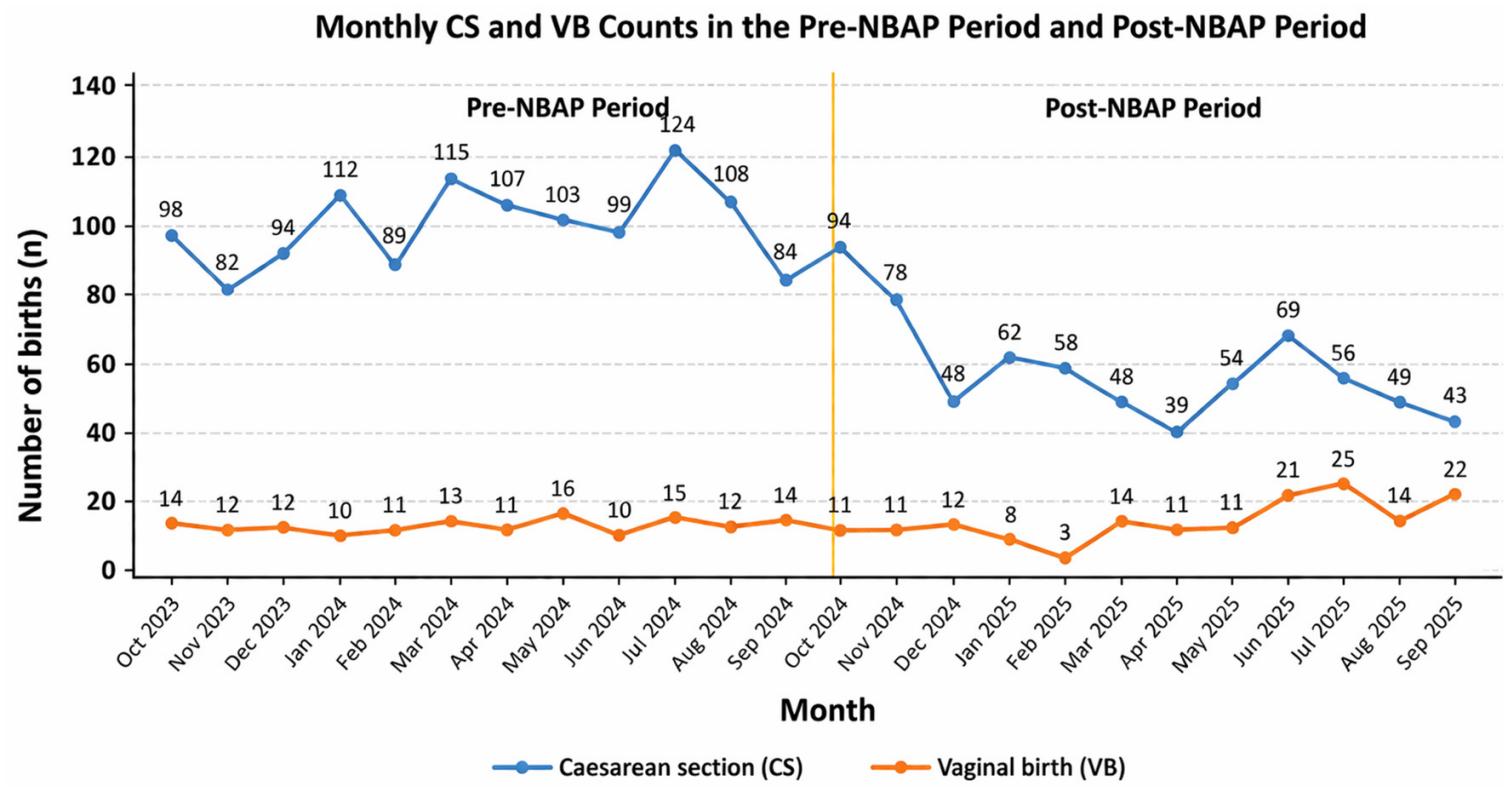
Monthly distribution of CS and VB before and after the implementation of the NBAP. The vertical line indicates the time of NBAP implementation (October 2024). A clear decline in CS counts and a gradual increase in VB are observed in the post-NBAP period.

As illustrated in Figure 1, CS remained the predominant mode of delivery throughout the study period. During the pre-NBAP phase, CS counts were consistently high, ranging from 82 to 124 per month, whereas VB remained relatively low and stable (10–16 per month).

Following the implementation of NBAP, a temporal change in delivery patterns was observed. CS counts declined from 94 in October 2024 to 42 in September 2025. In contrast, VB counts showed a gradual increase over time, ranging from 3 to 25 per month and peaking in July 2025 (*n* = 25).

Overall, these findings indicate a temporal association between NBAP implementation and a decrease in CS alongside an increase in VB; however, causality cannot be inferred due to the retrospective design of the study.

## Discussion

This study provides early evidence on changes in mode of birth following the implementation of the NBAP in a private hospital setting in Türkiye. Compared with the pre-NBAP period, the post-NBAP period was associated with lower CS rates and higher VB rates. A decrease was also observed in CS attributed to maternal request or fear of VB.

Globally, CS rates remain above levels recommended by the World Health Organization, particularly in private healthcare settings (1, 15). In Türkiye, CS rates are substantially higher than OECD averages, and private hospitals report especially high rates (3, 4). Robson-based analyses from Türkiye also support that repeat CS and maternal request contribute substantially to overall CS rates (5). NBAP was introduced as a national policy response aimed at reducing medically unnecessary CS while supporting safe birth practices (6, 12). The observed temporal changes in our cohort suggest that nationally implemented policies may coincide with measurable shifts in clinical patterns even in private-sector settings where CS is frequently preferred.

A substantial reduction in total birth volume (approximately 37%) was observed in the post-NBAP period. Several plausible explanations may account for this decline, including shifts in patient referral patterns, redistribution of deliveries across healthcare institutions, seasonal or demographic changes, and broader healthcare system dynamics. Additionally, private hospital utilization may have been influenced by economic factors, patient preferences, or policy-related changes occurring concurrently with NBAP implementation. Therefore, the observed changes in CS and VB should be interpreted with caution, as they may partly reflect changes in case volume rather than solely changes in clinical practice. Multicentre studies with longer follow-up will be important to disentangle policy-related temporal associations from background changes. Due to the retrospective design and lack of individual-level data on referral patterns or socioeconomic status, no statistical adjustment for this confounder could be performed.

Maternal request and fear of childbirth represented a substantial proportion of CS indications in the pre-NBAP period, consistent with prior evidence that psychosocial factors contribute to CS preference (9, 16, 17). The lower proportion observed post-NBAP may reflect increased exposure to counseling, changes in institutional practice, or heightened awareness regarding VB; however, causal inference cannot be established in this retrospective design. Evidence from prior studies indicates that antenatal education and individualized birth planning can improve childbirth perceptions and may increase VB rates (10, 18), and positive experiences with midwife-led care are associated with recommending VB in subsequent pregnancies (11).

Trends were generally consistent across age strata, including women aged 18–30 years. Despite these favorable shifts, VB rates remained well below international targets, indicating that sustained efforts—education, service redesign, and supportive clinical environments—are likely required for durable change (12, 15). Ethical considerations are also central: while reducing unnecessary CS is a public health goal, NBAP implementation should safeguard informed choice, maternal autonomy, and clinician professional judgment.

Although early improvements were observed, the sustainability of these changes remains uncertain. Long-term effectiveness of NBAP likely depends on continued education, institutional support, and alignment between policy goals and clinical practice. The gradual increase in VB during the post-NBAP period may suggest an emerging trend; however, longer follow-up is required.

Strengths of this study include the comparison of two consecutive 1-year periods within the same institution and the detailed categorization of CS indications. However, several limitations should be acknowledged. First, the retrospective single-centre design limits generalizability. Second, the absence of detailed socioeconomic, educational, and psychosocial variables restricts the ability to fully account for factors influencing mode of birth. Third, the substantial reduction in total birth volume represents a potential confounder that may have influenced the observed trends. Fourth, NBAP counseling status was based on clinical documentation, which may be subject to misclassification or incomplete recording. Finally, the before–after design does not allow causal inference, and the findings should be interpreted as temporal associations.

## Conclusion

In this private hospital cohort, the post-NBAP period was associated with a reduction in cesarean births and an increase in vaginal births, alongside a decrease in CS attributed to maternal request or fear of VB. Given concurrent changes in total birth volume, multicentre studies with longer follow-up and careful attention to maternal autonomy are needed to clarify and sustain the impact of NBAP-related strategies.

## Data Availability

The raw data supporting the conclusions of this article will be made available by the authors, without undue reservation.

## References

[1] Angolile CM Max BL Mushemba J Mashauri HL. Global increased caesarean section rates and public health implications: a call to action. Health Sci Rep. (2023) 6:e1274. doi: 10.1002/hsr2.1274 37216058 PMC10196217

[2] Galvão R Hawley NL da Silva CS Silveira MF. How obstetricians and pregnant women decide mode of birth in light of a recent regulation in Brazil. Women Birth. (2018) 31:e310–7. doi: 10.1016/j.wombi.2017.11.011 29229514

[3] Küçük M. Striking rise of caesarean section rates in Türkiye: there is a need for a whole new perspective. Pan Afr Med J. (2024) 48:1. doi: 10.11604/pamj.2024.48.6.12380 38946747 PMC11214144

[4] Topaktaş G Beylik U. Caesarean section rates in Türkiye: situation analysis and policy recommendations. Jinekoloji Obstetrik ve Neonatoloji Tip Dergisi. (2024) 21:102–13. doi: 10.38136/jgon.1482889

[5] Kinci MF Kasap B Akin MN Selimoglu B Taştan L Akin Gökbel D . (2024). Analysis of cesarean section ratios by Robson classification. J Obstetr Gynaecol India. 74:434–9. doi: 10.1007/s13224-023-01885-2 39568964 PMC11574227

[6] Republic of Türkiye Ministry of Health. Normal Birth Action Plan Promotion Meeting Held Under the Theme “Natural is Normal Birth”. (2023). Available online at: https://www.saglik.gov.tr/tr-105999/Dogal-Olan-Normal-Dogum-Temasiyla-Normal-Dogum-Eylem-Plani-Tanitim-Toplantisi-Yapildi.html (Accessed April 5, 2025)

[7] Ensari TA Kavak D Yirci B Elmas B Esin S Yalvac S . Women's preferences regarding the mode of delivery and current status of caesarean section in Türkiye. Jinekoloji Obstetrik ve Neonatoloji Tip Dergisi. (2022) 19:1–7. doi: 10.38136/jgon.1160913

[8] Eide KT Morken NH Bærøe K. Maternal reasons for requesting planned caesarean section in Norway: a qualitative study. BMC Pregnancy Childbirth. (2019) 19:102. doi: 10.1186/s12884-019-2250-6 30922267 PMC6440101

[9] Gökçe Isbir G Serçekuş P Yenal K Okumuş H Durgun Ozan Y Karabulut Ö . Prevalence and associated factors of fear of childbirth among Turkish pregnant women. J Reprod Infant Psychol. (2024) 42:62–77. doi: 10.1080/02646838.2022.2057938 35345941

[10] Ahmadpour P Moosavi S Mohammad-Alizadeh-Charandabi S Jahanfar S Mirghafourvand M. Effect of implementing a birth plan on maternal and neonatal outcomes: a randomized controlled trial. BMC Pregnancy Childbirth. (2022) 22:862. doi: 10.1186/s12884-022-05199-5 36419027 PMC9682672

[11] Aktaş S Küçük Alemdar D. Why mothers with midwifery-led vaginal births recommend that mode of birth: a qualitative study. J Reprod Infant Psychol. (2025) 43:1126–47. doi: 10.1080/02646838.2024.2328765 38466669

[12] Gaz S. Evaluation of the achievement level of strategic plans and targets related to mode of birth in Türkiye. Işletme Akademisi Dergisi. (2025) 6:57–70. doi: 10.1501/tarimbil_0000001016

[13] American College of Obstetricians and Gynecologists. Pregnancy at age 35 years or older. Obstet Gynecol. (2022) 140:348–66. doi: 10.1097/AOG.0000000000004873 35852294

[14] Schummers L Hacker MR Williams PL Hutcheon JA Vanderweele TJ McElrath TF . Variation in relationships between maternal age at first birth and pregnancy outcomes by maternal race: a population-based cohort study in the United States. BMJ Open. (2019) 9:e033697. doi: 10.1136/bmjopen-2019-033697 31843851 PMC6924831

[15] World Health Organization. Maternal Health and Child Health and Development. Geneva: WHO (2008). Available online at: http://www.who.int (Accessed October 27, 2022).

[16] Bohren MA Opiyo N Kingdon C Downe S Betrán AP. Optimising the use of caesarean section: a generic formative research protocol for implementation preparation. Reprod Health. (2019) 16:170. doi: 10.1186/s12978-019-0827-1 31744493 PMC6862737

[17] de Elejalde R Giolito E. A demand-smoothing incentive for caesarean deliveries. J Health Econ. (2021) 75:102411. doi: 10.1016/j.jhealeco.2020.102411 33341419

[18] Bal MD Yilmaz SD Beji NK. Care for evidence-based applications during pregnancy. Florence Nightingale J Nurs. (2013) 21:139–46.

